# Identifying predictors of vaccination willingness and attitudes during Covid-19: Machine learning multi-country study

**DOI:** 10.1192/j.eurpsy.2023.884

**Published:** 2023-07-19

**Authors:** M. Makhubela

**Affiliations:** University of Limpopo, Polokwane, South Africa

## Abstract

**Introduction:**

While there is some research that shows personal and psychological factors to be linked to disease-avoidant behaviour and attitudes in the time of Covid-19, this research is however mixed and inconsistent (i.e., some studies report a link and others do not).

**Objectives:**

In this study we clarify whether demographic and psychological factors specifically predict vaccination willingness and attitudes using Machine learning of a global survey sample from 137 countries (N = 24 000).

**Methods:**

Random forest machine learning algorithm was used to identify the strongest predictors of vaccination willingness and attitudes, while regression trees were developed to identify individuals at greater risk for anti-vaccination attitudes.

**Results:**

Conspiratorial thinking and lack of trust in experts were associated with vaccination attitudes and willingness.

**Conclusions:**

The findings underscore the role of conspiratorial beliefs in shaping the uptake of non-pharmacological and pharmacological novel pandemic protective measures.

**Disclosure of Interest:**

None Declared

